# Fibrin Clot Properties in Atherosclerotic Vascular Disease: From Pathophysiology to Clinical Outcomes

**DOI:** 10.3390/jcm10132999

**Published:** 2021-07-05

**Authors:** Michał Ząbczyk, Joanna Natorska, Anetta Undas

**Affiliations:** 1 John Paul II Hospital, 31-202 Kraków, Poland; michalzabczyk@op.pl (M.Z.); j.natorska@szpitaljp2.krakow.pl (J.N.); 2 Institute of Cardiology, Jagiellonian University Medical College, 31-202 Kraków, Poland

**Keywords:** atherosclerosis, coronary artery disease, fibrin clot, fibrinolysis, thromboembolism

## Abstract

Fibrin is a major component of thrombi formed on the surface of atherosclerotic plaques. Fibrin accumulation as a consequence of local blood coagulation activation takes place inside atherosclerotic lesions and contributes to their growth. The imbalance between thrombin-mediated fibrin formation and fibrin degradation might enhance atherosclerosis in relation to inflammatory states reflected by increased fibrinogen concentrations, the key determinant of fibrin characteristics. There are large interindividual differences in fibrin clot structure and function measured in plasma-based assays and in purified fibrinogen-based systems. Several observational studies have demonstrated that subjects who tend to generate denser fibrin networks displaying impaired clot lysis are at an increased risk of developing advanced atherosclerosis and arterial thromboembolic events. Moreover, the majority of cardiovascular risk factors are also associated with unfavorably altered fibrin clot properties, with their improvement following effective therapy, in particular with aspirin, statins, and anticoagulant agents. The prothrombotic fibrin clot phenotype has been reported to have a predictive value in terms of myocardial infarction, ischemic stroke, and acute limb ischemia. This review article summarizes available data on the association of fibrin clot characteristics with atherosclerotic vascular disease and its potential practical implications.

## 1. Introduction

Growing evidence indicates that the formation of denser fibrin networks, which are less susceptible to lysis, characterizes patients with atherosclerosis and arterial thromboembolic events. Several cardiovascular risk factors, such as hyperlipidemia, hypertension, smoking, or diabetes have also been shown to be associated with unfavorably altered fibrin clot properties in the general population. Low-dose aspirin, statins, better diabetes control, or smoking cessation have been shown to increase fibrin clot permeability and its susceptibility to lysis. Moreover, it has been shown that non-vitamin K antagonist oral anticoagulants (NOACs) are able to improve fibrin clot characteristics and contribute to the reduced risk of adverse clinical outcomes. The current review article summarizes available basic research and clinical papers deposited on PubMed over the last decade regarding associations between fibrin clot phenotype and atherosclerotic vascular disease, supported by the seminal papers from previous years. Moreover, data on novel therapeutic strategies, which can potentially influence fibrin clot characteristics, have been discussed.

## 2. Atherosclerotic Plaque Formation

Atherosclerosis is a major cause of cardiovascular disease that encompasses coronary artery disease, cerebrovascular disease, peripheral arterial disease, and aortic atherosclerosis. The current concept of the pathogenesis of atherosclerosis is based on chronic inflammation associated with modified lipid deposition and dysregulated immunity within the arterial wall [1,2,3]. The key driver of atherosclerosis is elevated low-density lipoprotein (LDL) prone to undergoing oxidative modification. Following endothelial cell injury with the subsequent influx of monocytes transformed into heterogeneous macrophages and other inflammatory cells, modified LDLs are extracellularly accumulated below the endothelium, leading to fatty streaks, an initial stage of plaque formation [2]. The formation of fibroatheroma and, finally, advanced atherosclerotic plaque is associated with the secretion of multiple chemoattractants and growth factors by leukocytes and arterial smooth muscle cells (SMCs) [2]. The proliferation of SMCs is associated with the production of large amounts of extracellular connective tissue matrix, including collagen, elastin, and proteoglycans [2,3]. Oxidized LDLs (oxLDLs) are taken up by immune cells within the atherosclerotic lesion with their subsequent transformation into foam cells, leading to plaque growth [4].

Neovascularization within the advanced plaque contributes to its gradual growth in part due to intraplaque hemorrhages. An increased density of microvessels has been found in ruptured atherosclerotic plaques [5], suggesting an important link between neovascularization and plaque instability [6]. Recent studies strongly suggest that plaque healing occurs in the natural course of atherosclerosis, with higher prevalence of healed plaques in patients with chronic manifestations of atherosclerotic vascular disease compared to those with recurrent acute coronary syndromes [7,8]. Unstable or vulnerable atherosclerotic plaques that show such characteristics as a thin fibrous cap, high macrophage content, high amounts of proinflammatory factors or a large necrotic core composed of foam cells and extracellular cholesterol [9] are prone to rupture, accompanied by occlusive thrombosis.

## 3. Blood Coagulation and Fibrin Formation in Atherosclerosis

The role of blood coagulation in the development and progression of atherosclerotic vascular disease reaches beyond thromboembolic complications. Macrophages can produce tissue factor (TF) [4], expressed also on microvesicles and its expression is regulated by inflammatory mediators, demonstrating the link between inflammation and thrombosis [10]. TF is the high-affinity receptor and cofactor for factor (F) VII/VIIa and the resultant TF-FVIIa complex activates FIX and FX [10]. A prothrombinase complex formed on activated platelets, including FXa, its cofactor FVa converts prothrombin to thrombin, the key enzyme of blood coagulation [11]. Pro-atherogenic actions of thrombin are associated with the activation of protease-activated receptors (PARs), leading to increased endothelial permeability, SMC migration and proliferation, and the activation of platelets and leukocytes, which promote vascular calcification and plaque development [11]. Activated platelets interact with leukocytes and stimulate them to release proinflammatory cytokines, reactive oxygen species, and provide the surface for the formation of tenase and prothrombinase complexes to generate thrombin from circulating prothrombin [10]. Fibrin formation occurs when minimal amounts of prothrombin have been activated (less than 5% of thrombin generation capacity) [8]. Fibrin accumulation within atherosclerotic plaque is involved in the disease progression, especially at the late stage of plaque formation [12]. Presence of fibrin within the necrotic core of damaged plaques supports its role in plaque growth and rupture [12,13]. Intraplaque fibrin has been shown to be more common in symptomatic than in asymptomatic atherosclerotic plaques [12]. Thrombin-activated FXIII, which covalently crosslinks fibrin fibers, also catalyzes the formation of intermolecular bonds between α2-antiplasmin, fibronectin, vitronectin, thrombospondin, and collagen, which in part explains fibrin accumulation with impaired fibrinolytic degradation within the lesions. Borissoff et al. [14] showed that ApoE-/-mice, which are prone to atherosclerosis, with genetically imposed 50% reduction in prothrombin were characterized by diminished atherosclerotic lesion formation and increased plaque stability, which suggests that coagulation activation is implicated in plaque development and progression and could be a potential therapeutic target. Thrombin promotes the accumulation of neutrophils and the production of reactive oxygen species, enhancing vascular inflammation. Of pivotal importance are observations made in 2010 suggesting that enhanced blood coagulation can be associated with plaque stability, given the fact that TF, FII, FX, and FXII activities are diminished along with plaque transformation to advanced stage [15]. Borissoff et al. [15] have also suggested that the loss of coagulation protein activity may contribute to the risk of plaque rupture.

Adventitial fibroblasts in normal arteries are able to express TF, while in atherosclerotic lesions, TF is also expressed by SMCs, foam cells, and macrophages, which can additionally release microparticle-derived TF [16]. TF was locally detected in 43% of patients with unstable coronary syndromes and in 12% of patients with stable coronary syndromes [17]. Moreover, about 40% higher blood levels of FVII have been reported in men with vulnerable atherosclerotic plaques in the coronary arteries, compared to those who had stable plaques [18]. The colocalization of several proteins involved in blood coagulation within the plaques, largely on macrophages, microvesicles, and SMCs [19], provides the rationale for the role of a local thrombin-mediated conversion of soluble fibrinogen into fibrin, the final product of blood coagulation, in the formation of atherosclerotic lesions.

Several studies have suggested that increased fibrinogen concentration, a key determinant of fibrin formation and its characteristics, is a risk factor for atherosclerotic vascular diseases, in particular coronary artery stenosis and myocardial infarction (MI) [20,21,22]. In the meta-analysis of 154,211 participants from 31 prospective studies, the hazard ratio (HR) for coronary heart disease and stroke was 1.78 (95% confidence interval [CI] 1.19–2.66) per 1 g/L increase in plasma fibrinogen concentrations [23]. The US National Health and Nutrition Examination Survey (NHANES) study showed that fibrinogen is associated with cardiovascular disease and about a 2.5-fold higher risk of all-cause and cardiovascular mortality during the 14 years of follow-up [24]. On the other hand, a Mendelian randomization study has shown no causal effect of fibrinogen on cardiovascular disease [25].

The localization of fibrin degradation products (i.e., D-dimer) within the human arterial wall suggests their potential atherogenic properties [26]. Higher D-dimer levels can be associated with atherosclerotic plaque remodeling or ongoing fibrinolysis [27]; however, both processes may trigger lipid deposition and modulate local inflammation within atherosclerotic plaques. Moreover, high levels of plasminogen activator inhibitor type 1 (PAI-1) have been identified both in the blood of coronary artery disease (CAD) patients and within unstable plaques [28]. Some genetically determined fibrinogen disorders, dysfibrinogenemias, have also been linked to atherosclerotic vascular disease and its thromboembolic manifestations, supporting the view that alterations to fibrin structure and function might be of greater importance than the fibrinogen concentration itself [29,30,31].

Fibrin acts as a scaffold for intravascular blood thrombi, enhancing platelet aggregation and thrombin generation, leading to a further increase in fibrin formation [29]. Fibrin(ogen) can interact with red blood cells through specific receptors, such as CD47, and with platelets (i.e., integrin αIIbβ3 or intercellular adhesion molecule 1) [29]. Cellular components embedded within the fibrin network after thrombus formation can modulate its properties. FXIII-dependent red blood cell retention in clots has been shown to impair the fibrin network structure, which delays thrombus degradation [32]. Scanning electron microscopic analysis of intracoronary thrombi obtained from patients with ST-segment elevation MI (STEMI) showed that fibrin content increased from about 30% to 80%, while red blood cell content decreased from 31% to 2% over time after the onset of chest pain [33,34]. It suggests that thrombus formation and its major component, fibrin, is a dynamic process [35,36]. Moreover, intravascular thrombi rich in red blood cells contain more neutrophils, reflecting a high thrombus burden, which has been shown to be associated with impaired reperfusion assessed at six months after the index event among patients with STEMI [35]. The presence of polyhedral erythrocytes, polyhedrocytes, has been linked with higher erythrocyte content, higher fibrinogen, and more significant stenosis in the culprit artery [36].

**Figure 1:**
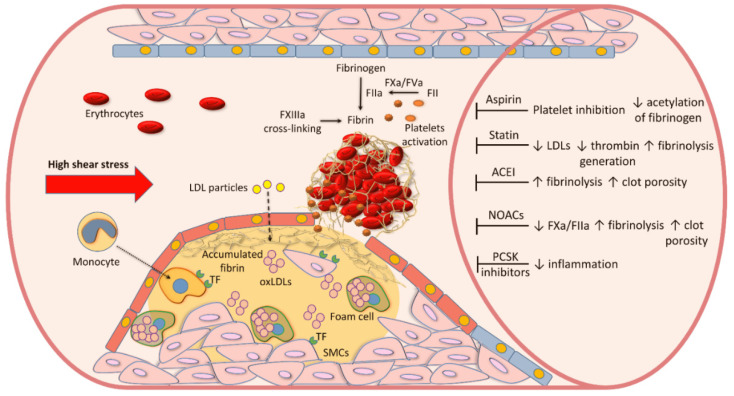
The initiation of an atherosclerotic lesion is associated with retention of low-density lipoproteins (LDL) and their oxidation (oxLDLs). oxLDLs stimulate recruitment of blood monocytes and their differentiation into macrophages. An uptake of oxLDLs by macrophages results in the formation of foam cells. Upon stimulation, vascular smooth muscle cells (SMCs) migrate and proliferate. Tissue factor (TF), expressed on macrophages and SMCs, is involved in coagulation activation, resulting in prothrombin (factor II, FII) conversion to thrombin (FIIa), which, in a prothrombinase complex with active FV (FVa), converts fibrinogen to fibrin. Several drugs, including aspirin, statins, angiotensin-converting enzyme inhibitors (ACEI), or non-vitamin K antagonist oral anticoagulants (NOACs) have been shown to modulate fibrin clot phenotype by different mechanisms. Proprotein convertase subtilisin kexin (PCSK) type 9 inhibitors are able to attenuate interplaque inflammation, but their effect on fibrin clot properties has not been reported yet. ↑—up-regulation, ↓—down-regulation.

A contribution of blood components, in particular key coagulation factors to atherothrombosis along with potential therapeutic targets, which can modulate fibrin clot structure and function are summarized in Figure 1.

## 4. Measures of Fibrin Clot Properties

**Figure 2:**
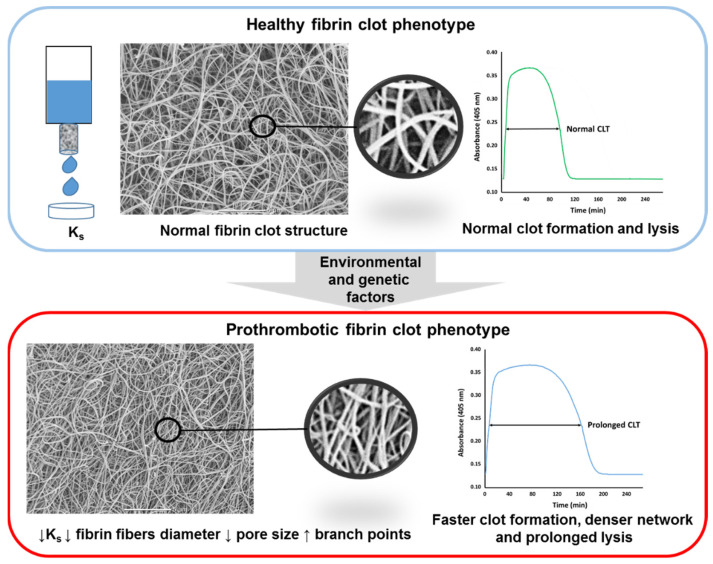
Fibrin clot structure differs between healthy persons and patients with atherosclerosis. A key measure describing plasma fibrin clot structure is its permeability. Reduced fibrin clot permeability (K_s_) is a typical feature of the prothrombotic fibrin clot phenotype, which is associated with lower fibrin fiber diameter, lower pore size area, and increased number of fibrin branch points. Faster clot formation results in denser fibrin network (indicated by higher clot turbidity), which is relatively resistant to lysis (prolonged clot lysis time; CLT). ↑—up-regulation, ↓—down-regulation.

Several parameters have been used in human subjects to assess fibrin clot properties. The structure of a fibrin clot generated from plasma (or purified fibrinogen) can be described using clot permeability (K_s_ or Darcy’s constant; reflecting volume of a buffer flowing through a fibrin gel during prespecified time) [37,38,39], turbidity (clot absorbance measured using a spectrophotometer at 405 or 340 nm) [40], or the direct measurement of fibrin fiber diameter, pore size, or fiber branching using microscopic techniques [41]. Fibrin clot susceptibility to lysis is measured by turbidimetry using several assays with either exogenous tissue plasminogen activator or plasmin added to clotted plasma [40,42,43]. A so-called prothrombotic fibrin clot phenotype encompasses reduced K_s_ associated with typical changes in fibrin structure, such as lower fibrin fibers diameter, lower pore size area between particular fibers, and an increased number of branch points, along with faster fibrin formation and prolonged lysis time (Figure 2).

## 5. Cardiovascular Risk Factors

The majority of well-established cardiovascular risk factors which have been reported to be associated with prothrombotic fibrin clot properties are presented in Table 1 [44,45,46,47,48,49,50,51,52,53,54]. Of note, there is controversy around the association of unfavorably altered clot properties and hypercholesterolemia. Low HDL cholesterol has been reported to be associated with more prothrombotic clot features [55].

## 6. Coronary Artery Disease

### 6.1. Acute MI

Acute MI is a leading cause of mortality in high-income countries and is a major thrombotic manifestation of atherosclerotic lesions in coronary arteries [56]. Dense fibrin networks, as evidenced by reduced K_s_ and impaired clot susceptibility to lysis, have been reported in patients with acute MI, at least in part associated with increased oxidative stress and the extent of inflammation [57]. Prolonged clot lysis time has been confirmed as a risk factor for MI in men and women in a case–control study performed on 800 acute MI patients and 1123 controls [58]. It has been hypothesized that unfavorably modified fibrin clot properties observed in acute MI are also driven by increased thrombin generation and platelet activation, expressed by a release of large amounts of proteins affecting clot features, for instance beta-thromboglobulin and platelet factor 4 [34]. In a cohort of 421 men with acute MI compared to 642 controls, hypofibrinolysis has been associated with the risk of a first MI in young men, but not in subjects aged ≥ 50 years, and CLT strongly correlated with body mass index [44]. As expected, patients with acute coronary events compared to stable coronary artery disease were characterized by a more prothrombotic fibrin clot phenotype, as reflected by lower K_s_ and prolonged lysis time, related to a higher body mass index, higher blood pressure and higher C-reactive protein levels [57]. Platelet-derived factors, such as P-selectin of platelet-factor 4, exert a similar effect, promoting prothrombotic fibrin clot features [59]. Serum levels of P-selectin and soluble CD40 ligand were also positively associated with thrombus fibrin content [34].

It has been demonstrated that fibrin is the main constituent (60%) of intracoronary thrombi obtained during thrombectomy in acute STEMI (within 12 h since the symptom onset), with amounts increasing with time. Increased intracoronary fibrin content has also been found to be positively associated with denser plasma fibrin networks, reflected by reduced K_s_ [36], which indicates that plasma clot characteristics have an impact on fibrin formed in intravascular thrombi. Recently, it has been suggested that intracoronary thrombi may have another type of fibrin on the surface. We have identified a thin layer of a fibrin biofilm on the surface of 15% intracoronary thrombi from acute MI patients, which was solely associated with higher plasma fibrinogen levels [60]. Heparin infusion during coronary angiography and thrombectomy probably reduces this proportion. This observation provided additional ex vivo evidence supporting the findings of Macrae and colleagues [61], who have shown that fibrin forms a film connected to the clot network and covers whole blood clots, which may protect against infiltration by bacteria or viruses, with potential impact on wound healing and thrombus fragmentation. Despite that there are no available reports describing the presence of fibrin biofilm on thrombi obtained from other locations. The relevance of the biofilm formation in human thrombosis requires further investigation.

It is unclear whether fibrin clot composition differs among patients with the same condition. Proteomics data has shown that clot-bound protein composition can influence fibrin properties [62]. A preliminary shotgun proteomic analysis performed on plasma clots from four patients during acute MI and two months later revealed time-dependent changes in the clot structure, which may influence clot stability and its susceptibility for lysis [63]. Differences in fibrinolysis proteins, such as increased amounts of α2-antiplasmin, in acute MI may at least in part explain time-dependent changes in the clot structure following MI [63].

From a practical point of view, the issue of prognostication based on plasma fibrin clot characteristics appears to be of vital importance. Growing evidence indicates that the prothrombotic fibrin phenotype can predict cardiovascular events. A PLATO substudy performed on 4354 patients following acute MI has shown that a validated turbidimetric assay employed to assess plasma clot lysis time and clot maximum turbidity at hospital discharge, while on dual antiplatelet therapy, is able to predict adverse clinical outcomes during a 12 month follow-up period [64]. After adjustment for cardiovascular risk factors, each 50% increase in lysis time was associated with a 1.17-times higher risk of cardiovascular death or MI, and a 1.36-fold higher risk of cardiovascular death alone. A similar increase in plasma clot maximum turbidity was associated with a 1.24-fold increased risk of death (hazard ratio 1.24, 95% confidence interval 1.03–1.50) [64]. Fibrin clot density and resistance to lysis increased with higher levels of N-terminal pro B-type natriuretic peptide (NT-proBNP) and troponin T, which are known to be associated with a greater inflammatory response. The authors concluded that higher NT-proBNP levels can be associated with worse outcomes as a consequence of impaired fibrin clot features, which may lead to an increased risk of atherothrombosis [64]. Even after additional adjustment for leukocyte count, high-sensitivity C-reactive protein, high-sensitivity troponin T, cystatin C, NT-proBNP, and growth differentiation factor-15 levels, the association with death remained significant solely for lysis time [64]. It remains to be established whether clot density, or permeability, could have a similar prognostic value.

### 6.2. Stable CAD

Stable CAD, defined as angina pectoris, MI history, or presence of atherosclerotic plaques, has been associated with unfavorably modified fibrin clot properties [65]. Reduced fibrin clot lysability has been reported in asymptomatic women with present coronary plaque determined by computed tomography angiography, compared to both women without plaque and men, suggesting a sex-dependent link between coronary atherosclerosis and prolonged clot lysis time [66]. Patients with symptomatic CAD formed clots with more rigid structures and increased fibrin fiber mass-to-length ratio [67]. A history of MI in 33 young patients with documented CAD was associated with prothrombotic fibrin clot characteristics, including increased clot stiffness and a slower fibrinolysis rate, compared to healthy controls [68]. Increased lipoprotein(a) levels, a well-established risk factor for premature atherosclerosis, have been shown to alter fibrin clot properties and were associated with reduced K_s_ and prolonged clot lysis time in patients with a history of MI [69]. Moreover, in advanced CAD patients, lipoprotein(a), a genetically determined risk factor for premature atherosclerosis, levels predicted K_s_ but not lysis time [70]. Similarly, type 2 diabetes concomitant to CAD was associated with prolonged clot lysis time and a more compact fibrin clot structure compared to non-diabetic CAD patients [71]. The rs495828 risk allele within the ABO locus, which is known to be associated with an increased risk of MI in CAD patients, has also been shown to be associated with a more compact fibrin network structure, as evidenced by higher clot maximum absorbance, but not lysis time, among 773 stable CAD patients [72]. In a long-term follow-up study, the area under the curve of turbidimetrically monitored clot formation and lysis predicted future cardiovascular events in stable CAD (HR = 2.4, 95%CI 1.2–4.6) [73]. Altogether, CAD is associated with the prothrombotic clot phenotype governed by several largely environmental factors.

## 7. Peripheral Arterial Disease

Prothrombotic fibrin clot properties were observed for the first time in 2009 in patients with intermittent claudication, a typical manifestation of peripheral arterial disease (PAD) [74,75] which occurs in 18–20% in individuals over 70 years of age, including the most serious presentation, limb ischemia, affecting up to 3% of patients with PAD. [76]. The group from Leeds reported that unfavorably altered fibrin clot structure and function are detectable in apparently healthy close relatives of patients with claudication [74,75]. In 106 PAD patients, there was a reduction by 20% in K_s_ when compared to controls, and it was associated with 31% prolonged CLT; the two alterations in fibrin properties predicted PAD progression during long-term follow-up [77]. Similarly, 13.4% reduced K_s_ with no difference in CLT was found in patients with a history of acute lower limb ischemia compared to individuals without any history of such event [77]. Interestingly, premature PAD has also been identified as the clinical condition associated with a less favorable fibrin clot phenotype, in particular 30% lower clot permeability, compared to normal conditions, and no difference in the phenotype is observed in typical older PAD patients [78]. Moreover, in critical limb ischemia patients, who represent up to 3% of patients with PAD, restenosis detected within one year following endovascular therapy was associated with slightly reduced K_s_ and prolonged CLT at baseline, accompanied by elevated thrombin generation and von Willebrand factor antigen; however, the fibrin clot variables cannot predict re-intervention, amputation, and death during further 3-year follow-up [79].

## 8. Aortic Aneurysm

Scott et al. [80] have shown that patients with abdominal aortic aneurysm (AAA) form denser fibrin clots with smaller pore sizes which are more resistant to lysis. Such prothrombotic clot phenotype was associated with the size of aneurysm and may play a role in its development. A further study performed on 169 AAA patients, including about 40% with a history of stable angina or MI, showed that plasma levels of D-dimer and thrombin-antithrombin (TAT) complexes were independent predictors of AAA growth rate [81]. An increase in D-dimer level by 500 ng/mL, or TAT level by 1 µg/mL was associated with enlargement of the aneurysm size by 0.21 and 0.24 mm per year, respectively. However, it is not known to date whether prothrombotic fibrin clot phenotype in patients with aneurysm may contribute to clinical outcomes in particular its rupture or rapid enlargement.

## 9. Pharmacological Treatment and Fibrin Clot Properties

### 9.1. Cholesterol-Lowering Agents

Statins (3-hydroxy-methylglutaryl coenzyme A reductase inhibitors) may exert several cholesterol-independent antithrombotic effects, including the down-regulation of TF expression and enhanced protein C activation via increased endothelial thrombomodulin expression [82]. Although data linking hypercholesterolemia with prothrombotic clot characteristics are limited and unconvincing, statins (simvastatin or atorvastatin at a dose of 40 mg/day for 4 weeks) used in patients with stable CAD to effectively reduce LDL cholesterol have been shown to reduce plasma clot density, reflected by higher K_s_ and shortened CLT, despite no impact on plasma fibrinogen levels [83]. Favorable effects of statins on fibrin clot structure and function were supported by the study in which a 3-month use of simvastatin (40 mg/d) led to a slight, though significant, increase in K_s_ and a shortened clot lysis time in patients with LDL cholesterol < 3.4 mmol/L free of clinically evident CAD, like in the JUPITER trial with rosuvastatin [84]. This effect appeared to be associated with a decrease in CRP concentrations, suggesting links between the antithrombotic and anti-inflammatory effects of statins.

Novel therapeutic strategies to lower LDL cholesterol based on fully humanized monoclonal antibodies that bind free plasma proprotein convertase subtilisin/kexin type 9 (PCSK9) [85], different cholesteryl ester transfer protein (CETP) inhibitors [86], and antisense oligonucleotides targeting apolipoprotein(a) [87,88] are currently under investigation. To our knowledge, their potential effects on fibrin clot properties have not been investigated yet.

### 9.2. Aspirin

Aspirin was the first effective antiplatelet therapy for the prevention of ischemic events in patients with atherosclerotic vascular disease. Aspirin treatment has been shown to be associated with the formation of thicker fibrin fibers and improved clot susceptibility to lysis in stable CAD patients [89]. The mechanism of aspirin action on fibrin clot structure is unclear; however, fibrinogen acetylation [90,91] has been postulated as a major contributor despite controversies as to whether aspirin at therapeutic doses (75–150 mg/day) might exert such effects observed largely in vitro. Interestingly, low-dose aspirin (75 mg/day) has been shown to exert a stronger effect on fibrin clot properties than 320 mg/day [92,93]. In ten stable CAD patients treated with aspirin at a dose of 75 mg/day, aspirin withdrawal was associated with 32% reduced K_s_ after one week and 41% reduced K_s_ after two weeks when compared to values observed during treatment [94]. It is unclear to what extent fibrin-related mechanisms might add to the well-known antithrombotic effect caused by cyclooxygenase-1 inhibition and antiaggregatory effects on platelets.

### 9.3. Angiotensin-Converting Enzyme Inhibitors (ACEI)

Antihypertensive therapy with ACEI has been found to modulate fibrin clot properties in association with reduced complement component C3 levels [54]; however, similar effects were noted for other agents lowering blood pressure. In a double-blind study performed in men aged < 70 years with a history of MI or hospitalization for unstable angina, a four-week treatment with quinapril was associated with a K_s_ increase by 13% and shortening of clot lysis time by 28% [83]. It has been suggested that the effect of ACEIs on increased fibrin clot porosity can be associated with reduced plasma fibrinogen levels [95].

### 9.4. NOACs

NOACs, including rivaroxaban and apixaban, which are selective and direct FXa inhibitors, and dabigatran as a direct thrombin inhibitor, are used to prevent and treat thromboembolic events [96]. Clots formed from normal plasma spiked with rivaroxaban (174 ng/mL) or apixaban (128 ng/mL) at therapeutic levels resulted in a less dense and more permeable clot structure with thicker fibers [97]. Varin et al. [98] have demonstrated more permeable fibrin networks composed of thicker fibrin fibers in plasma spiked with rivaroxaban at a concentration of 0.15 μg/mL, which was in line with the findings of Janion-Sadowska et al. [99] made in plasma-based assays in patients 2–6 h after rivaroxaban intake (20 mg/day). A seminal COMPASS trial has shown that rivaroxaban use at a dose of 2.5 mg twice daily combined with 100 mg of aspirin can prevent cardiovascular death in patients with advanced cardiovascular disease, mostly represented by those with stable CAD [100,101]. It has also been shown that pharmacological inhibition of FXa promotes the regression of advanced atherosclerotic plaques and enhances plaque stability in mice treated with rivaroxaban (1.2 mg/g) for 14 weeks [102], suggesting that the inhibition of FXa may be beneficial both in the prevention and regression of atherosclerotic vascular disease by down-regulated activation of PARs. The influence of low-dose rivaroxaban on fibrin clot phenotype still remains to be elucidated; however, based on the current knowledge, it can be assumed that rivaroxaban 2.5 mg bid might improve fibrin properties like rivaroxaban 20 mg/day, though to a smaller extent, and thus contributing to reduced risk of adverse clinical outcomes largely thromboembolic by nature.

## 10. Clinical Implications

The formation of dense fibrin networks which are relatively resistant to lysis has been observed in patients with atherosclerotic vascular disease, in particular CAD and PAD, along with those who experience arterial thromboembolism. The prothrombotic features of fibrin clots that are largely determined by environmental factors can be improved by the control of cardiovascular risk factors, in particular the normalization of glycemia and statin use. Growing evidence suggests that measures of clot characteristics, such as clot permeability and clot lysis time, may predict arterial thromboembolic events. Therefore, fibrin clot measures could serve as prognostic markers in patients at risk of arterial thromboembolism. However, there is a need for large studies to validate the available observations and standardization of the assays used to characterize the clot phenotype, though the first step initiated by the scientific subcommittee of the International Society on Thrombosis and Haemostasis has been made to implement the measurement of K_s_ and CLT in clinical practice [43,103].

## 11. Conclusions

Taken together, several studies demonstrated that the formation of more compact fibrin networks displaying lower susceptibility to lysis are implicated in the progression of atherosclerosis and the occurrence of thromboembolic manifestations, in particular MI (see Table 2). The observations might support accumulating data on clinical benefits from the use of anticoagulant agents in the prevention of cardiovascular mortality. It remains to be established whether any specific modulators of fibrinolytic efficiency might be useful in the prevention of clinical outcomes in atherosclerotic vascular disease, still the major cause of morbidity and mortality worldwide.

## Figures and Tables

**Table 1:** Cardiovascular risk factors in association with fibrin clot properties.

Cardiovascular Risk Factor	Study Design	No. of Subjects	Measure	Reference
Age	Case-control	642 controls (and 421 MI patients)	No clear effect on CLT in controls	[44]
	Cohort study	80 healthy controls	↑ CLT and Lys50 with increasing age	[45]
	Cohort study	2010 healthy controls	↑ clot turbidity and CLT with increasing age	[46]
	Cross-sectional study	2000 healthy controls	No clear effect on CLT	[47]
Body-mass index (BMI)	Cohort study	1288 healthy subjects	BMI positively associated with CLT in men and women	[48]
Family history of coronary artery disease	Case-control	100 healthy male relatives of patients with premature coronary artery disease and 100 healthy controls	↓ K_s_ and ↑ clot turbidity in relatives of patients	[39]
Current smoking	Case-control	642 controls (and 421 MI patients)	No clear effect on CLT	[44]
	Cross-sectional study	2000 healthy controls	No clear effect on CLT	[47]
	Case-control	34 healthy male smokers and 34 nonsmokers	↑ clot strength, ↑ clot turbidity, ↓ fibrin fiber diameter in smokers	[49]
	Case-control	44 male cigarette smokers and 44 nonsmokers	↓ K_s_ and ↑ clot lysis time	[50]
	Cohort study	30 healthy subjects	No clear effect on CLT	[51]
Lipid profile	Cohort study	30 healthy subjects	Low-density lipoprotein cholesterol level positively associated with CLT	[51]
Diabetes	Case-control	642 controls (and 421 MI patients)	No clear effect on CLT	[44]
	Case-control	150 patients with type 2 diabetes and 50 controls	↓ K_s_ and ↑ clot turbidity associated with glycated hemoglobin levels	[52]
	Interventional study	20 type 2 diabetes subjects	↑ K_s_ after achievement of glycemic control; K_s_ associated with glycated hemoglobin levels	[53]
Arterial hypertension	Cohort study	61 patients with essential arterial hypertension	↑ K_s_, ↓ clot lysis time, ↓ clot resistance to lysis at 6 months of antihypertensive treatment	[54]

Myocardial infarction (MI), clot lysis time (CLT), fibrin clot permeability (K_s_), ↑—up-regulation, ↓—down-regulation.

**Table 2:** Table summarizing the recent data on plasma fibrin clot properties, including fibrin clot permeability (K_s_) and clot lysis time (CLT) in atherosclerotic patients.

Author (Year of Publication)	Study Type	Sample Size, Condition	Main Findings
Sadowski et al. (2014) [34]	Cohort study	40 acute MI patients	Plasma levels of platelet activation markers correlated with thrombus fibrin content
Zalewski et al. (2015) [36]	Cohort study	80 acute MI patients	Low K_s_ was independently associated with high fibrin content within the intracoronary thrombi
Sumaya W et al. (2018) [64]	Cohort study	4354 acute coronary syndrome patients	Prolonged lysis time was associated with cardiovascular death/MI
Ramanathan R et al. (2018) [66]	Cross-sectional study	138 individuals without known cardiovascular disease	Women with coronary plaques had reduced fibrin clot lysability compared to women or men without coronary plaques
Neergaard-Petersen S et al. (2014) [71]	Cohort study	581 CAD patients, including 148 subjects with type 2 diabetes	Type 2 diabetes in CAD patients was associated with prothrombotic fibrin clot compared to non-diabetic CAD patients
Winther-Larsen A et al. (2020) [72]	Cohort study	773 patients with stable CAD	The *ABO* risk allele (rs495828) was associated with a more compact fibrin network in stable CAD patients
Neergaard-Petersen S et al. (2020) [73]	Cohort study	786 patients with stable CAD	Increased area under the curve of clot formation and lysis predicted cardiovascular events
Karpińska I et al. (2020) [77]	Case-control study	43 patients with a history of acute limb ischemia, 43 patients with cryptogenic stroke, 43 controls	Increased clot density and hypofibrinolysis characterized patients with acute limb ischemia compared to controls
Nowakowski et al. (2019) [79]	Case-control study	85 patients with critical limb ischemia and restenosis and 47 PAD patients	Restenosis compared to PAD was associated with reduced K_s_ and prolonged CLT
Scott DJ et al. (2011) [80]	Case-control study	42 patients with large AAA, 40 patients with small AAA, and 49 controls	Patients with AAA compared to controls formed denser plasma clots, which were more resistant to lysis

Myocardial infarction (MI), coronary artery disease (CAD), peripheral arterial disease (PAD), abdominal aortic aneurysm (AAA).
